# Draft Genome Sequences of Two Extensively Drug-Resistant *Acinetobacter baumannii* Strains Isolated from Pus Samples

**DOI:** 10.1128/genomeA.00161-16

**Published:** 2016-03-24

**Authors:** Niranjana Mahalingam, Bhavani Manivannan, Sudhir Jadhao, Gayathri Mishra, Pravin Nilawe, Bulagonda Eswarappa Pradeep

**Affiliations:** a Department of Microbiology, Sri Sathya Sai Institute of Higher Medical Sciences, Prasanthigram, India; b Department of Biosciences, Sri Sathya Sai Institute of Higher Learning, Prasanthi Nilayam, India; c Bionivid technology Pvt. Ltd., Kasturi Nagar, Bengaluru, India; d Thermo Fisher Scientific, Mumbai, India

## Abstract

We report the draft genomes of two extensively drug-resistant (XDR) *Acinetobacter baumannii* strains isolated from pus samples of two patients with surgical site infections at Sri Sathya Sai Institute of Higher Medical Sciences, Prasanthigram, India. The average genomic size and G+C content are 4 Mbp and 38.96% (AB28) and 4 Mbp and 38.94% (AB30), respectively.

## GENOME ANNOUNCEMENT

*Acinetobacter baumannii* is a Gram negative, nonlactose fermenting bacillus known to cause a variety of community acquired and nosocomial infections including meningitis, endocarditis, septicemia, and ventilator-associated pneumonia while exhibiting enhanced resistance to several classes of antibiotics (1, 2). Here we report the draft genome sequences of two extensively drug-resistant (XDR) nosocomial *Acinetobacter baumannii* strains, AB28 and AB30.

The strain AB28 was isolated from a 37-year old male patient with polytrauma. It was cultured from the pus sample of surgical site infection at 12-days post fracture while AB30 was cultured from the pus of an 80-year old female patient, who underwent repair of intertrochanteric fracture. On the 13th post-operative day, there was surgical site infection of the skin and subcutaneous tissue. Both of these patients were admitted in the same department at the same time and had orthopedic implants done as part of fracture repair. The pus samples were received on the same day.

Vitek-2 revealed that the strains AB28 and AB30 are resistant to aminoglycosides (gentamicin), carbapenems (imipenem, meropenem), fluoroquinolones (ciprofloxacin, levofloxacin), penicillin and beta lactamase inhibitors (piperacillin-tazobactam, cefoperazone-sulbactam), extended spectrum cephalosporins (ceftazidime, cefepime), trimethoprim-sulfamethoxazole, and were found to be susceptible to tigecycline, minocycline, and colistin.

The genomes of AB28 and AB30 were sequenced using an Ion PGM (personal genome machine) 400 kit and chip 318. Sequencing generated 1,195,415 reads with a mean length of 273.67 ± 110.35 bp for AB28 and 1,192,947 reads with an average length of 263.85 ± 118.86 bp for AB30. *De novo* assembly was performed using SPADES v3.5.0 (3). The AB28 draft genome has 97 contigs for an estimated total size of 4,007,105 bp with G+C content of 38.96% and *N*_50_ contig size of 3,852,879 bp. The AB30 draft genome has 88 contigs encompassing a total sequence of 4,007,203 bp with G+C content of 38.94 % and *N*_50_ contig size of 3,869,138. Annotation by RAST (4) and NCBI PGAAP servers of the draft genomes reveals that AB28 has 3,891 genes with 2,794 coding sequences, 3 rRNAs, 42 tRNAs, and 1 noncoding RNA (ncRNA) while AB30 has 3,886 genes with 2,579 cDNAs, 3 rRNAs, 41 tRNAs, and 1 ncRNA.

No plasmid sequence was identified when analyzed by Webcutter (v2.0) and Plasmid Finder (5) in both the draft genomes. Analysis by Resfinder v2.1 (6) indicates the presence of multiple antibiotic resistance genes against beta-lactams, aminoglycosides, macrolides and sulfonamides in the sequenced genomes. Antibiotic-resistant genes, namely, β-lactam (blaOXA-23, blaOXA-58, blaOXA-66, blaCARB-2, blaNDM-1, blaPER-1, and blaADC-25), sulfonamide (sul1 and sul2), macrolide (mph[E] and msr[E]), aminoglycoside (strA, strB, armA, and aph[3′]-VIb) genes were found in both the genomes. PHAST server (7) analysis has identified 3 putative prophage sequences in AB28 and 2 putative prophage sequences in AB30 genomes. The draft genomes reported here would provide basis for deeper insights into the mechanisms of nosocomial infections by XDR *A. baumannii*.

### Nucleotide sequence accession numbers.

The draft genome sequences have been deposited in DDBJ/EMBL/GenBank under the accession numbers LQBT00000000 (AB28) and LQBV00000000 (AB30). The versions described in this article are the ﬁrst versions.
